# Serological evidence of avian HEV antibodies in apparently healthy chickens in southwest Nigeria

**DOI:** 10.1371/journal.pone.0247889

**Published:** 2021-02-26

**Authors:** Fisayo Temilade Osamudiamen, Olusola Aanuoluwapo Akanbi, Daniel Oladimeji Oluwayelu, C. -Thomas Bock, Patrycja Klink

**Affiliations:** 1 Department of Veterinary Microbiology, University of Ibadan, Ibadan, Nigeria; 2 Department of Infectious Diseases, Viral Gastroenteritis and Hepatitis Pathogens and Enteroviruses, Robert Koch Institute, Berlin, Germany; 3 Institute of Tropical Medicine, University of Tuebingen, Tuebingen, Germany; CEA, FRANCE

## Abstract

Avian hepatitis E virus (aHEV) is associated with hepatitis-splenomegaly syndrome, big liver and spleen disease and hepatic rupture haemorrhage syndrome. However, the knowledge about aHEV in commercial layer chickens in Nigeria is scarce. In this study, 460 serum samples obtained from 36 apparently healthy commercial layer chicken flocks in three states (Ogun, Osun and Oyo States) of southwestern Nigeria were analysed by enzyme linked immunosorbent assay for the presence of anti-aHEV immunoglobulin Y (IgY) antibodies. In total, the overall seroprevalence of anti-aHEV antibodies was 14.6%. The serological analysis revealed that 75% of the flocks examined were positive for anti-aHEV IgY antibodies from chickens of various ages in all three states. The percentage of the seropositive chickens in the three states varied from flock to flock ranging from 60% to 88.8% and seropositive chickens were detected at any age (24–52 weeks of age) without significant differences between the age groups. This is the first report assessing the presence of aHEV antibodies in chickens from Nigeria. The detection of anti-aHEV antibodies in commercial layer chickens in this study emphasizes the importance of serosurveillance in disease monitoring due to the economic threat posed by aHEV as a result of decreased egg production and increased mortality in affected commercial layer chicken farms. However, further studies are essential to reveal the clinical implications and to assess the real burden of aHEV in Nigeria.

## Introduction

Avian hepatitis E virus (aHEV) is classified into the *Orthohepevirus B* species of the family *Hepeviridae* and *(Orthohepevirus B* species) comprises of HEV isolated from chickens and wild birds [1]. HEV infections may occur without obvious clinical signs and symptoms in many domestic animal species, but in chickens it occurs both subclinically and with certain pathognomonic signs. Infections with the virus have been proven to be the main causative agent of hepatitis-splenomegaly syndrome (HSS) and big liver and spleen disease (BLSD) in laying hens and broiler breeders [2, 3]. Recently, aHEV infections have also been associated with hepatic rupture haemorrhage syndrome (HRHS) in chickens [4, 5]. Besides the markedly enlarged spleen and liver, both ovarian regression and presence of serosanguinous abdominal fluid or massive haemorrhages in the abdominal cavity are frequently associated with the HSS and BLSD [2, 6–8]. Also, increased mortality and a decreased egg production have been reported for HSS as well as BLSD [9, 10]. However, aHEVs have also been detected in apparently healthy chickens without HSS symptoms [11, 12]. The virus appears to spread easily within and among flocks through the faecal-oral route transmission; no other routes of transmission have been demonstrated in natural or experimental avian models [7, 13].

Serological evidences show that aHEV is widespread in chicken flocks with reported seropositive rates of 71% in the United States of America, 90% in Spain, 20% in Brazil, 57% in Korea and 56% in Poland [3, 9, 14–16]. However, in Nigeria, despite reports of serological prevalence of HEV in domestic animals e.g. pigs, sheep and goats [17–19], the knowledge about aHEV in chickens is scarce. Until so far, only one publication reported the presence of aHEV RNA in 6.7% of chicken droppings in Nigeria [20] but no seroprevalence studies have been performed. In view of the fact that aHEV infection can be subclinical and accounts for economic losses incurred in poultry production, this study was designed to assess the possible circulation of aHEV in chicken flocks in three selected states (Ogun, Osun and Oyo States) which are considered as a major hub of commercial poultry production in southwestern, Nigeria [21].

## Materials and methods

### Samples and sampling locations

**Fig 1:**
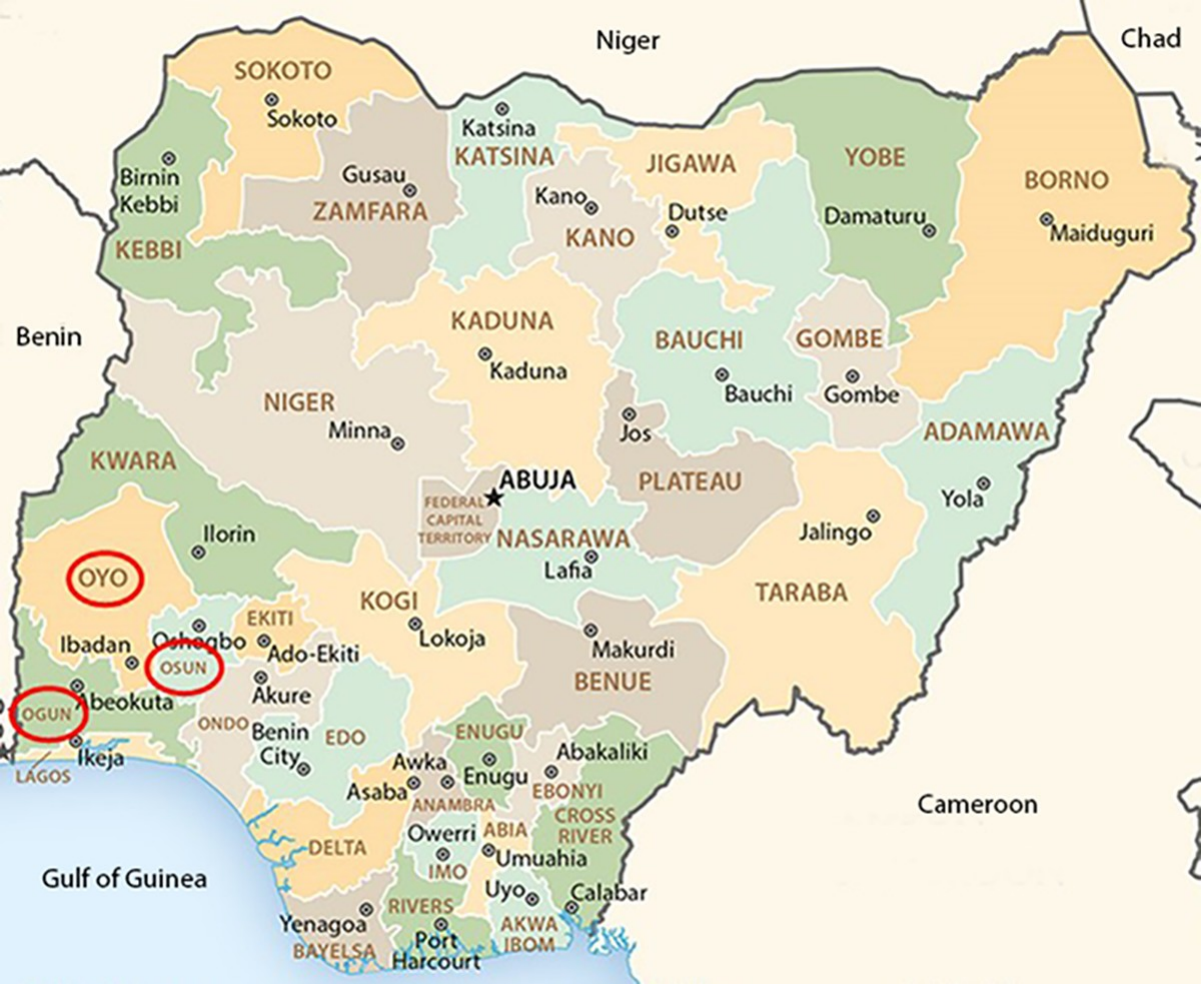
Sample locations in southwest Nigeria. Federal states of Nigeria and their boundaries are depicted by different colors, the national capital Abuja by star, and state capitals by black circles. Red circles depict the sample location states Oyo, Osun and Ogun. (Nigeria administrative map adapted from: https://www.cia.gov/library/publications/resources/cia-maps-publications/Nigeria.html (public domain)).

A cross sectional study was performed. Depending on the flock size, between 8 and 26 chickens were sampled and tested in a flock. However, the average number of chickens sampled per flock was 13. In total, 460 apparently healthy layer chickens farmed on battery cage management system were sampled from 36 different commercial poultry farms located within three states (Ogun, Osun and Oyo States) in southwestern Nigeria with different production profiles and were tested for anti-aHEV IgY antibodies. The number of flocks sampled per state was representative of the number of layer chicken flocks in the Nigerian states. While 125 serum samples were collected from 10 farms in Ogun State, 124 serum samples were collected from 9 farms in Osun State and 211 serum samples were collected from 17 farms in Oyo State (Fig 1). Serum samples were collected between October 2018 to October 2019 from apparently healthy chickens aged between 24 to 52 weeks without any clinical signs suggestive of aHEV.

The study protocol was reviewed and approved by the Animal Care and Use Research Ethics Committee (ACUREC) University of Ibadan with approval number UI-ACUREC /17/0042.

### Sample collection and preparation

Blood samples (2 ml each) were collected from the jugular veins of the chickens into sterile plain tube and transported on ice. The blood was left to coagulate at room temperature overnight and thereafter centrifuged at 8000 rpm for 1 minute to separate serum. All serum samples were then kept frozen at -80°C until November 2019 when aliquots were thawed and spotted on Whatman™ 903 protein saver cards (Cytiva, formerly GE Healthcare Life Sciences, Marlborough, USA). Thirty (30) μl of serum was applied slowly to one pre-printed dotted line on a filter spot of labeled Whatman™ 903 protein saver cards. Five (5) dried serum spots (DSS) were made per sample, the filter cards were air dried and kept in a zip lock plastic bag together with two desiccant bags and closed tightly. The zip lock plastic bags were also labeled appropriately and stored at -20°C until shipment at room temperature to Robert Koch Institute, Berlin, Germany.

### Detection of anti-avian HEV IgY antibodies in chicken sera by ELISA

Two DSS were punched out from the Whatman^™^ cards for each sample and put into a 1.5 ml labelled safe-lock tube. Therefore, the spots were cut out without excess filter paper and the tweezers and puncher used were cleaned with 70% ethanol after each sample. Phosphate-buffered saline (PBS) solution for elution was prepared using 500 ml PBS, 15 ml foetal calf serum, and 250 μl Tween 20. 500 μl of PBS elution buffer was added to the reaction tube to completely cover the two filter spots and incubated in the refrigerator (2–8°C) overnight. The eluate (~450 μl) was transferred into a new, labelled reaction tube and tested for anti-aHEV IgY antibodies by enzyme linked immunosorbent assay (ELISA) using the Big Liver and Spleen Disease Antibody test kit (BLS CK 131, BioChek, Berkshire, United Kingdom) according to the manufacturer’s protocol. The BioChek ELISA test kit used in this study is highly sensitive (98%) to detect avian HEV antibodies in chicken serum with a specificity of > 98% which has been validated in previous publications [16, 22, 23]. High titre positive and negative/reference controls for the detection and validation of aHEV antibodies were from the BioChek ELISA kit and used according to the manufacturer’s instructions.

### Statistical analysis

Statistical analysis was performed using Fishers exact test in the SPSS software version 16 (SPSS Inc. Released 2007. SPSS for Windows, Version 16.0. Chicago, SPSS Inc., USA).

## Results

### Seroprevalence of anti-aHEV IgY in commercial layer chickens in Nigeria

In total, 14.6% of all tested serum samples (n = 67/460) were positive for aHEV IgY antibodies. Twenty-seven (75%) of the 36 commercial layers flocks tested positive for anti-aHEV IgY antibodies from chickens of various ages in three states. The median age of the analysed chickens was 42 weeks (interquartile range, IQR: 32–47 weeks). The percentage of the seropositive chickens varied from flock to flock ranging from 60.0% to 88.8% and seropositive chickens were detected at any age (Table 1).

**Table 1:** Distribution of anti-aHEV antibodies in commercial layers in Nigeria based on age.

Age in weeks (median ± IQR)	Flocks tested, N	Positive flocks, N (%)	Serum samples tested, N	Positive serum samples, N (%)
24–30 (25 ± 4)	6	5 (83.3)	96	14 (14.6)
31–45 (40 ± 4)	15	11 (73.3)	180	25 (13.9)
46–52 (48 ± 4)	15	11 (73.3)	184	28 (15.2)
**Total**	**36**	**27 (75.0)**	**460**	**67 (14.6)**

IQR: Interquartile range.

In the different age groups, chickens younger than 30 weeks, between 31–45 weeks and older than 46 weeks showed an anti-aHEV seropositivity of 14.6%, 13.9% and 15.2%, respectively (Table 1). No statistically significant association of anti-aHEV seropositivity with age could be observed.

From the 460 samples (36 flocks) studied, 18 chickens (n = 125) from 6/10 flocks, 19 chickens (n = 124) from 8/9 flocks and 30 chickens (n = 211) from 13/17 flocks tested positive for aHEV IgY antibodies in Ogun, Osun and Oyo States, respectively (Table 2).

**Table 2:** Distribution of anti-aHEV antibodies in commercial layers in Nigeria based on sampling location.

Farm location (State)	Flocks tested, N	Positive flocks, N (%)	Serum samples tested, N	Positive serum samples, N (%)
Ogun	10	6 (60.0)	125	18 (14.4)
Osun	9	8 (88.8)	124	19 (15.3)
Oyo	17	13 (76.5)	211	30 (14.2)
Total	36	27 (75.0)	460	67 (14.6)

In Ogun State, a flock seroprevalence of 60.0% (n = 6/10) was observed and 14.4% (n = 18/125) of the serum samples were positive for anti-aHEV IgY antibodies, while in Osun State, the flock seroprevalence was 88.8% (n = 8/9) and 15.3% of the serum samples were anti-aHEV IgY antibody positive (n = 19/124). In Oyo State, the flock seroprevalence was 76.5% (n = 13/17), while 14.2% of the serum samples had detectable anti-aHEV IgY antibodies (n = 30/211) (Table 2).

There was observed variation in the anti-aHEV seroprevalence among the three states studied. The population size (flock density) of the commercial layer chickens in each state was used as criterion for the choice of the number of flocks sampled per state. The number of flocks sampled per state is representative of the number of commercial layer chicken flocks per state. However, there was no significant difference between the numbers of positive samples detected from the three states.

## Discussion

Avian hepatitis E (aHEV) virus infection has been reported worldwide in poultry flocks [2, 9, 24, 25]. In Africa, the presence of aHEV antibodies has been detected in chickens in Egypt with an overall seroprevalence of 15.7% [23]. In Nigeria, previous studies have shown evidence for the presence of HEV antibodies in domestic animals in Nigeria, such as pigs with a prevalence of 55.6% [18], 32.8% [19] and 57.4% [17] as well as sheep and goats with prevalence of 10.5% and 37.2%, respectively [19]. But there has been no evidence for the detection of aHEV antibodies among poultry in Nigeria despite the occurrence of hepatitis splenomegaly syndrome and drastic reduction in egg production accompanied with high morbidity and mortality reported at a time in some layer farms (I. Akpan, personal communication, March 9, 2016).

In this study, serum samples were collected from commercial layer chickens from three states (Ogun, Osun and Oyo states), which have been chosen because they are considered as the main hub of poultry production in Nigeria [21]. Layer chickens contribute to the economic development of the West African sub region and the country because of the income generated from egg and chicken production. Hence, drops in egg production and increased mortality which are associated with apparently healthy chickens infected with avian HEV [26] would lead to economic losses for the poultry farmers.

The high anti-aHEV seropositivity rates of flocks (75%) and chickens (14.6%) detected in our study indicates that aHEV infection is endemic and widespread in Nigerian poultry population despite the fact that no previous outbreak of aHEV disease has been reported in the country. This finding is consistent with previous reports from Spain, which reported that 89.7% of chicken flocks were positive for aHEV [9]. Furthermore, in USA and Taiwan a comparably high seroprevalence in chicken flocks and chickens has been observed, with seropositivity of 71% and 30% in the USA [3] and 95.08% and 40.57% in Taiwan [25] respectively. According to Hsu and Tsai [23], this high aHEV seropositivity rate suggested the possibility of aHEV transmission from asymptomatic cases or repeated introduction through an unknown common source(s). However, a lower seroprevalence has been reported from Poland (56.1% and 21.3% [16], and Korea (57% and 28%) [15]. The anti-aHEV seroprevalence observed among flocks and chickens in the three states is not significantly different, which may be due to the fact that the states are contiguous to each other and all are located in the southwest geographical region of Nigeria.

An age-related difference of seropositivity in chickens in this study could not be observed as the seroprevalence of chickens older than 46 weeks of age (15.2%) was comparable to chickens between 31–45 weeks of age (13.9%) and those aged younger than 31 weeks (14.6%). This is contrary to a previous report in which seropositivity of 36% was reported in adult chickens older than 18 weeks as compared with 17% in younger chickens [3]. The commercial ELISA test kit used is dedicated to detect IgY antibodies to avian HEV in serum of chickens and included positive and negative controls. However, an age-related association of aHEV seroprevalence is discussed controversially, as there are also studies reporting higher seroprevalence in chickens younger than 30 weeks of age in comparison to older chickens [27, 28]. As a limitation of our study the DSS used in this study were transported at room temperature to the laboratory after freezing of the initial sample and the DSS. Freeze-thawing of samples might affect the results of the ELISA [29, 30]. Although other studies report no significant change after several freeze-thaw cycles [31], it cannot be excluded that some samples were misclassified as false-positive or false-negative. Likewise, we cannot exclude false-positive samples and further studies are needed to clarify the presence of aHEV in chickens in Nigeria. The convenience sampling method employed in the selection of chickens could also introduce some statistical bias affecting the results as the selected group might not be representative of the general chicken population.

This study represents the first detection of antibodies to aHEV in commercial layer chickens in Nigeria and sub-Saharan Africa.

## Conclusion

Our study reported a widespread seroprevalence of aHEV in apparently healthy commercial layer chicken flocks in three states in Nigeria. Although, the chickens sampled were apparently healthy with no obvious clinical signs, this study which is the first in Nigeria, further establishes the reports of decreased egg production by poultry farmers in the study areas. It could be inferred that there is a natural subclinical transmission of aHEV among the commercial layer chickens in Nigeria, as the southwestern states are considered to be the main hub of poultry production in Nigeria. The detection of anti-aHEV antibodies in commercial chickens is important as a serosurveillance tool in aHEV disease monitoring. This is because chicken meat and eggs are a major source of animal protein for the Nigerian populace. The presence of anti-aHEV antibodies is also an indirect indicator of circulating aHEV in poultry. This also confirms the existence of aHEV in Nigeria and justifies the need for constant serosurveillance. Further studies are needed especially in other regions of Nigeria to evaluate the economic burden, spread and risk factors of aHEV infection, and preventive measures to curb its spread.

## References

[1] Sun P, Lin S, He S, Zhou EM, Zhao Q. Avian Hepatitis E Virus: With the Trend of Genotypes and Host Expansion. Front Microbiol. 2019;10:1696. 10.3389/fmicb.2019.01696 31396195 PMC6668596

[2] Haqshenas G, Shivaprasad HL, Woolcock PR, Read DH, Meng XJ. Genetic identification and characterization of a novel virus related to human hepatitis E virus from chickens with hepatitis-splenomegaly syndrome in the United States. J Gen Virol. 2001;82(Pt 10):2449–62. 10.1099/0022-1317-82-10-2449 11562538

[3] Huang FF, Haqshenas G, Shivaprasad HL, Guenette DK, Woolcock PR, Larsen CT, et al. Heterogeneity and seroprevalence of a newly identified avian hepatitis e virus from chickens in the United States. J Clin Microbiol. 2002;40(11):4197–202. 10.1128/jcm.40.11.4197-4202.2002 12409397 PMC139663

[4] Su Q, Li Y, Meng F, Cui Z, Chang S, Zhao P. Hepatic rupture hemorrhage syndrome in chickens caused by a novel genotype avian hepatitis E virus. Vet Microbiol. 2018;222:91–97. 10.1016/j.vetmic.2018.06.019 30080679

[5] Su Q, Zhang Z, Zhang Y, Cui Z, Chang S, Zhao P. Complete genome analysis of avian hepatitis E virus from chicken with hepatic rupture hemorrhage syndrome. Vet Microbiol. 2020;242:108577. 10.1016/j.vetmic.2020.108577 32122587

[6] Payne CJ, Ellis TM, Plant SL, Gregory AR, Wilcox GE. Sequence data suggests big liver and spleen disease virus (BLSV) is genetically related to hepatitis E virus. Vet Microbiol. 1999;68:119–25. 10.1016/s0378-1135(99)00067-x 10501168

[7] Yugo DM, Hauck R, Shivaprasad HL, Meng XJ. Hepatitis Virus Infections in Poultry. Avian Dis. 2016;60(3):576–88. 10.1637/11229-070515-Review.1 27610716

[8] Thiry D, Mauroy A, Pavio N, Purdy MA, Rose N, Thiry E, et al. Hepatitis E Virus and Related Viruses in Animals. Transbound Emerg Dis. 2017;64(1):37–52. 10.1111/tbed.12351 25919649 PMC7169709

[9] Peralta B, Biarnes M, Ordonez G, Porta R, Martin M, Mateu E, et al. Evidence of widespread infection of avian hepatitis E virus (avian HEV) in chickens from Spain. Vet Microbiol. 2009;137(1–2):31–6. 10.1016/j.vetmic.2008.12.010 19136224

[10] Sun ZF, Larsen CT, Dunlop A, Huang FF, Pierson FW, Toth TE, et al. Genetic identification of avian hepatitis E virus (HEV) from healthy chicken flocks and characterization of the capsid gene of 14 avian HEV isolates from chickens with hepatitis–splenomegaly syndrome in different geographical regions of the United States. J Gen Virol. 2004;85:693–700. 10.1099/vir.0.19582-0 14993655

[11] Yugo DM, Meng XJ. Hepatitis E virus: food borne, waterborne and zoonotic transmission. Int J Enviro Res Public Health. 2013;10:4507–33. 10.3390/ijerph10104507 PMC3823334 24071919

[12] Zhang X, Bilic I, Troxler S, Hess M. Evidence of genotypes 1 and 3 of avian hepatitis E virus in wild birds. Virus Res. 2017;228:75–78. 10.1016/j.virusres.2016.11.028 27890632

[13] Meng XJ. From barnyard to food table: the omnipresence of hepatitis E virus and risk for zoonotic infection and food safety Virus Res. 2011;161(1):23–30. 10.1016/j.virusres.2011.01.016 21316404 PMC3126906

[14] Vitral CL, Pinto MA, Lewis-Ximenez LL, Khudyakov YE, dos Santos DR, Gaspar AM. Serological evidence of hepatitis E virus infection in different animal species from the Southeast of Brazil. Mem Inst Oswaldo Cruz. 2005;100(2):117–22. 10.1590/s0074-02762005000200003 16021297

[15] Kwon HM, Sung HW, Meng XJ. Serological prevalence, genetic identification, and characterization of the first strains of avian hepatitis E virus from chickens in Korea. Virus Genes. 2012;45(2):237–45. 10.1007/s11262-012-0761-6 22639103

[16] Matczuk AK, Ćwiek K, Wieliczko A. Avian hepatitis E virus is widespread among chickens in Poland and belongs to genotype 2. Arch Virol. 2019;164:595–99. 10.1007/s00705-018-4089-y 30392050 PMC6373257

[17] Oluwayelu D, Adebiyi A, Abiola J, Akingbola T, Cadmus S. Serologic evidence of hepatitis E virus activity among slaughtered pigs and in selected pig farms in Ibadan, Nigeria: implications for zoonotic transmission? Tropical Vet. 2017;35:91–100.

[18] Owolodun OA, Gerber PF, Giménez-Lirola LG, Kwaga JK, Opriessnig T. First report of hepatitis E virus circulation in domestic pigs in Nigeria. American J Trop Med Hygiene. 2014;91:699–704. 10.4269/ajtmh.14-0144 25002299 PMC4183390

[19] Junaid SA, Agina SE, Jaiye K. Seroprevalence of Hepatitis E virus among domestic animals in Plateau state, Nigeria. British Microbiol Res J. 2014;4:924–34. 10.4137/VRT.S15422 25512696 PMC4251053

[20] Ogbolu EE, Obi RK. Detection of Hepatitis E Virus RNA in Chicken Droppings and Pig Feces in Ogun and Lagos States, South Western, Nigeria. J Adv Med Medl Res. 2016;1:8.

[21] Owoade AA, Oluwayelu DO, Fagbohun OA, Ammerlaan W, Mulders MN, Muller CP. Serologic Evidence of Chicken Infectious Anemia in Commercial Chicken Flocks in Southwest Nigeria. Avian Dis. 2004;48:202–05. 10.1637/7075 15077816

[22] Troxler S, Pac K, Prokofieva I, Liebhart D, Chodakowska B, Furmanek D, et al. Subclinical circulation of avian hepatitis E virus within a multiple-age rearing and broiler breeder farm indicates persistence and vertical transmission of the virus. Avian Pathol. 2014;43(4):310–8. 10.1080/03079457.2014.924616 24828493

[23] Sultan S, Osman N. Serological evidence for the presence of infectious avian hepatitis E virus among chicken flocks in Egypt. Egyptian J Virol. 2016;13:27–33.

[24] Handlinger JH, Williams W. An egg drop associated with splenomegaly in broiler breeders. Avian Dis. 1988;32:773–78. 3202773

[25] Hsu IWY, Tsai HJ. Hepatitis E virus in chickens, Taiwan, 2013. Emerg Infect Dis. 2014;20:149. 10.3201/eid2001.131224 24378180 PMC3884734

[26] Sprygin AV, Nikonova ZB, Zinyakov NG. Avian hepatitis E virus identified in Russian chicken flocks exhibits high genetic divergence based on the ORF2 capsid gene. Avian Pathol. 2012;41(5):459–63. 10.1080/03079457.2012.711464 22967203

[27] Gerber PF, Trampel DW, Opriessnig T. Identification and characterization of avian hepatitis E virus in 2013 outbreaks of hepatitis-splenomegaly syndrome in two US layer operations. Avian Pathol. 2014;43(4):357–63. 10.1080/03079457.2014.935755 25010035

[28] Zhao Q, Sun Y, Zhao J, Hu S, Zhao F, Chen F, et al. Development and application of an indirect ELISA for detection of antibodies against avian hepatitis E virus. J Virol Methods. 2013;187(1):32–6. 10.1016/j.jviromet.2012.08.026 23000753

[29] Boadella M, Gortazar C. Effect of haemolysis and repeated freeze-thawing cycles on wild boar serum antibody testing by ELISA. BMC Res Notes. 2011;4:498. 10.1186/1756-0500-4-498 22087883 PMC3226466

[30] Kurian A, Neumann EJ, Hall WF, Marks D. Effects of blood sample mishandling on ELISA results for infectious bronchitis virus, avian encephalomyelitis virus and chicken anaemia virus. Vet J. 2012;192(3):378–81. 10.1016/j.tvjl.2011.08.028 22015139

[31] Pinsky NA, Huddleston JM, Jacobson RM, Wollan PC, Poland GA. Effect of multiple freeze-thaw cycles on detection of measles, mumps, and rubella virus antibodies. Clin Diagn Lab Immunol. 2003;10:19–21. 10.1128/cdli.10.1.19-21.2003 12522034 PMC145292

